# Appetite Control Might not Be Improved after Weight Loss in Adolescents with Obesity, Despite Non-Persistent Metabolic Syndrome

**DOI:** 10.3390/nu12123885

**Published:** 2020-12-18

**Authors:** Valérie Julian, Laurie Isacco, Marwa Khammassi, Alicia Fillon, Maud Miguet, Frederic Dutheil, Daniel Courteix, Marek Zak, Jacek Bicki, Stanisław Głuszek, Martine Duclos, Yves Boirie, Bruno Pereira, David Thivel

**Affiliations:** 1 Department of Sport Medicine and Functional Explorations, Clermont-Ferrand University Hospital, G. Montpied Hospital, 63000 Clermont-Ferrand, France; vjulian@chu-clermontferrand.fr (V.J.); mduclos@chu-clermontferrand.fr (M.D.); 2 UFR Medicine, Clermont Auvergne University, 63000 Clermont-Ferrand, France; yves.boirie@inrae.fr; 3 EA 3533, Laboratory of the Metabolic Adaptations to Exercise under Physiological and Pathological Conditions (AME2P), Clermont Auvergne University, 63000 Clermont-Ferrand, France; laurie.isacco@uca.fr (L.I.); khammassimarwa.issep@hotmail.com (M.K.); fillonalicia@gmail.com (A.F.); maud.miguet@gmail.com (M.M.); daniel.courteix@uca.fr (D.C.); 4 Departement de Médecine du travail, Clermont-Ferrand University Hospital, G. Montpied Hospital, 63000 Clermont-Ferrand, France; fdutheil@chu-clermontferrand.fr; 5 CNRS, LaPSCo, Physiological and Psychosocial Stress, Clermont Auvergne University, 63000 Clermont-Ferrand, France; 6 Collegium Medicum, Jan Kochanowski University, Zeromskiego 5, 25-369 Kielce, Poland; mkzak@ujk.edu.pl (M.Z.); jackibicki@wp.pl (J.B.); sgluszek@wp.pl (S.G.); 7 INRA, UMR 1019, 63000 Clermont-Ferrand, France; 8 CRNH-Auvergne, 63000 Clermont-Ferrand, France; 9 Department of Human Nutrition, Clermont-Ferrand University Hospital, G. Montpied Hospital, 63000 Clermont-Ferrand, France; 10 Clermont-Ferrand University Hospital, Biostatistics unit (DRCI), 63000 Clermont-Ferrand, France; bpereira@chu-clermontferrand.fr

**Keywords:** pediatric obesity, metabolic syndrome, energy intake, appetite control

## Abstract

The aim of this study was to evaluate the effect of a multidisciplinary weight loss intervention on energy intake and appetite sensations in adolescents with obesity, depending on the initial diagnosis or persistence of the metabolic syndrome. Ninety-two adolescents with obesity (12–15 years) followed a 16-week multidisciplinary weight loss intervention. Anthropometric and body composition characteristics, metabolic profile, ad libitum daily energy intake, and appetite sensations were assessed before and after the intervention. The presence of metabolic syndrome (MS) was determined at baseline (MS vs. non-MS) and after the program (persistent vs. non-persistent). While the intervention was effective in inducing weight loss (body weight T0: 87.1 ± 14.9 vs. T1: 81.2 ± 13.0 kg; *p* < 0.001) and body composition improvements in both adolescents with and without MS, energy intake (*p* = 0.07), hunger (*p* = 0.008), and prospective food consumption (*p* = 0.03) increased, while fullness decreased (*p* = 0.04) in both groups. Energy intake and appetite were not improved in non-persistent MS after the program and remained significantly higher among non-persistent adolescents compared with initially non-MS adolescents. To conclude, appetite control seems impaired in obese adolescents, irrespective of being affected by MS or not, whereas the treatment of MS in this population might fail to effectively preclude the adolescents from potential post-intervention compensatory food intake and subsequent weight regain.

## 1. Introduction

Pediatric obesity is an alarming public health concern with one out of five children suffering from obesity in Europe [1]. While adolescents with obesity have an 80% increased risk of remaining obese once adult [2] and of developing associated metabolic disorders that might increase their morbidity and reduce their life expectancy [3], the development of effective anti-obesity strategies remains a priority.

Multidisciplinary interventions combining physical activity, nutritional guidelines, and psychological support are actually recommended [4] and have been shown to be effective (at least in the short term) in inducing significant body weight and body composition [5], physical fitness [5], cardio-metabolic profile [6], and health-related quality of life [7] improvements, among others. Some recent results have indicated, however, that adolescents with obesity increase their daily energy intake in response to such interventions [8,9,10], which might contribute to the usually-observed post-treatment weight regain. While the role played by adolescents’ cognitive profiles has been established to partly account for these appetitive responses to weight loss, the potential implications of their cardio-metabolic profiles remain beyond any doubt.

Taetzsch et al. recently found a positive and significant association between the unhealthy metabolic profile of adult women with obesity and food craving [11], which is in line with previous results underlying a similar association between metabolic syndrome (MS) and unhealthy eating behaviors in children and adolescents [12]. Despite their scarcity, some studies tried to explore the potential mechanisms linking metabolic disorders and impaired appetite control and unhealthy eating habits. In their work, Farr et al. observed an hypo-activation of the food reward system in adults diagnosed with MS, accompanied by an increased energy intake [13]. According to their results, the higher the number of MS components, the lower the reward-related caudate and amygdala activation to food cues [13]. Similarly, Anthony et al. reported impaired appetite control in adult men with central and peripheral insulin resistance [14]. Importantly, some recent research also described alterations of the concentrations and regulations of some key physiological actors involved in the control of appetite (ghrelin, peptide YY_3-36_, or cholecystokinin) in adults with obesity diagnosed with a metabolic syndrome [15,16]. Altogether, these studies suggest a potential negative impact of the metabolic syndrome in the control of energy intake and appetite that needs to be further explored.

To date, we have been unable to find any evidence regarding the potential association between appetite control and the diagnosis of MS in adolescents with severe obesity, and whether the initial presence or persistence of MS after weight loss would differently affect energy intake and appetite in this population.

The aim of the present study was thus to evaluate the effect of a 16-week multidisciplinary weight loss intervention on energy intake and appetite sensations in adolescents with obesity, initially diagnosed or not with MS. We also compared these appetitive responses to this intervention between adolescents with persistent and non-persistent MS by the end of the program.

## 2. Population and Methods

### 2.1. Population

Ninety-two adolescents with obesity (12–15 years) took part in this study, and 83 adolescents (62 girls) completed the study (any drop-outs were due to family and academic reasons). The adolescents were recruited from an inpatient pediatric obesity center (La Bourboule, France). To be recruited, the adolescents had to be (i) aged 12 to 15 years; (ii) overweight/obese, in compliance with applicable national criteria; and (iii) Tanner 3–4; (iv), with no contraindications to physical exercise. The present analyses rest on the combination of data from two studies previously conducted by our team, using the same methodologies and pursued within the same center among adolescents with similar baseline characteristics (Study 1: Bioethics Committee Ref. No 2015-A01024-45, Clinical trial NCT02626273; Study 2: Bioethics Committee Ref. No 2017-A00817-46, Clinical trial NCT03466359). Any potential study effects were tested on the outcome studied, and since no effects were observed, data pooling from both studies was deemed feasible. Similarly, no gender effect was observed, and the data from both boys and girls were pooled. All experiments were conducted in full compliance with the Declaration of Helsinki, and written informed consent was obtained from all adolescents and their legal guardians.

### 2.2. Study Design

All the adolescents attended a medical examination with a pediatrician and then completed baseline anthropometric and body composition evaluations (dual X-ray absorptiometry (DXA)). A fasting blood sample was drawn to determine lipid profile, insulinemia, and glycemia, and blood pressure (BP) was measured. On a separate occasion, they were asked to join the laboratory to perform a full day of nutritional evaluation (energy intake and appetite). Participants then joined the local pediatric obesity center for a 16-week inpatient multidisciplinary weight-management intervention. All measurements were performed at baseline (T0) and after 4 months of intervention (T1).

### 2.3. Measurements

Anthropometric measures and body composition.

Body weight, height, and waist and hip circumference (WC and HC) were measured following the usual clinical recommendations. Body mass index (BMI) was calculated using weight (kg)/height (m^2^). Age and sex specific BMI percentiles (97th) for the French population were used to define obesity. Body composition (fat mass percentage (FM%) and fat-free mass (FFM) in kg) was measured by DXA (QDR4500A, Hologic, Waltham, MA, USA).

#### 2.3.1. Blood Pressure

Systolic (SBP) and diastolic (DBP) blood pressure were assessed after 20 min of rest according to appropriate clinical guidelines, with the subject in the sitting position with uncrossed legs, feet flat on the floor, and left arm supported above heart level. SBP and DBP were recorded using a mercury sphygmomanometer, using a cuff size appropriate to the patient’s arm circumference.

#### 2.3.2. Blood Samples

Blood samples were drawn by an experienced nurse by venipuncture after an overnight fast of 12 h. Samples were centrifuged (at 4000× *g* for 10 min at 4 °C), and plasma was stored at −80 °C until analysis. Plasma glucose and total cholesterol concentrations were determined by enzymatic methods (Modular P900; Roche Diagnostics, Meylan, France). Plasma insulin concentration was measured by chemiluminescence (Immulite2000, Diagnostic Products Corporation, Los Angeles, CA, USA). Insulin resistance was determined using the homeostasis model assessment index for insulin resistance (HOMA-IR = fasting insulin level (mIU/L) × fasting glucose level (mmol/L)/22.5). LDL cholesterol (LDL-C), HDL cholesterol (HDL-C), and triglyceride (TG) concentrations were assessed by the enzymatic colorimetric method.

#### 2.3.3. Detection of the Metabolic Syndrome

MS was diagnosed according to the criteria proposed by Chen and collaborators for children and adolescents [17] using thresholds adapted to the French population and considering the presence of three or more of the following criteria: BMI ≥ 97th percentiles for age and sex; SBP or DBP ≥ 90th percentile; HDL-C ≤ 0.4 g·L^−1^ or TG ≥ 1 g·L^−1^ in youth <10 years and ≥1.3 g·L^−1^ in youth >10 years; and fasting glucose ≥ 1.1 g·L^−1^ or HOMA-IR > 75th percentile. This method has been previously used in similar populations [18,19].

#### 2.3.4. Ad libitum Energy Intake

At 08:00, after an overnight fast, the adolescents were offered a standardized calibrated breakfast that respected the recommendations for their age (≈500 kcal) [20]. Lunch and dinner meals were served ad libitum using a buffet-type meal. As previously described [8], the content of the buffet was determined based on the adolescent’s food preferences and eating habits. Top rated items as well as disliked ones and items liked but not usually consumed were excluded to avoid over-, under-, and occasional consumption. At lunch, the menu was composed of beef steak, pasta, mustard, cheese, yogurt, apple sauce, fruits, and bread. The dinner menu was composed of ham/turkey, beans, mashed potatoes, cheese, yogurt, apple sauce, fruits, and bread. Food items were presented in abundance and accompanied with tap water only. Adolescents made their choices and composed their trays individually before joining their habitual table (5 adolescents per table). Adolescents were told to eat until feeling comfortably satiated and had access to extra food if wanted. Food intake was weighted by the experimenters, and the macro nutritive distribution (proportion of fat, carbohydrates (CHO) and protein) as well as the total energy consumption in kcal were calculated using the software Bilnut 4.0. This methodology has been previously validated and published [21]. Between the two ad libitum test meals, the adolescents were requested not to engage in any moderate to vigorous physical activity and mainly performed sedentary activities such as reading, homework, or board games.

#### 2.3.5. Subjective Appetite Sensations

Appetite sensations were collected throughout the day using visual analogue scales (150 mm scales). Adolescents had to report their hunger, fullness, desire to eat (DTE), and prospective food consumption (PFC) at six regulated times: before and right after breakfast, lunch, and dinner. The questions were as follows: (i) “How hungry do you feel?”, (ii) “How full do you feel?”, (iii) “Would you like to eat something?”, (iv) “How much do you think you can eat?” (adolescents were asked to respond on a scale from “not at all” to “a lot”). This method has been previously validated [22].

#### 2.3.6. Multidisciplinary Weight Loss Program

The 4-month inpatient multidisciplinary weight loss program combined physical activity, nutritional education, and psychological support. The physical activity intervention was composed of four supervised 60-min physical activity sessions per week including aerobic training, strength training, aquatic activities, and leisure-time activities. Concomitantly, the adolescents attended 2 h of physical education per week at school. The adolescents also attended nutritional education classes twice a month led by a dietician and received psychological support through individualized consultations with a professional once a month. During the intervention, the adolescents were submitted to a controlled normo-caloric diet based on their age and sex recommendations [23]. The inpatient nature of the intervention guaranteed compliance to the diet and physical activity interventions as well as to the psychological and educative interventions.

### 2.4. Statistical Analysis

Statistical analyses were performed using Stata software (Version 15, StataCorp, College Station, TEXAS USA). Continuous data were expressed as mean and standard-deviation. The assumption of normality was assessed using the Shapiro–Wilk test. The intra-group comparisons between baseline (T0) and T1 for continuous variables were carried out using paired Student *t*-test or Wilcoxon test when the assumptions of *t*-tests were not met. The study of homoscedasticity was studied using Pitman’s test of equality of variance. These analyses were realized in the whole sample and in adolescents with persistent and non-persistent MS. Then, to evaluate the intervention effect between adolescents initially diagnosed with or without the MS and to measure the effect between persistent and non-persistent MS (i.e., to compare baseline vs. T1 change between groups), random-effects models for repeated data were performed. A participant was considered as a random-effect in order to measure between and within adolescent variability, whereas group (with or without MS, persistent and non-persistent MS), time (T0 and T1), and group x time interactions were fixed effects, and gender was an adjustment variable due to a possible confounding effect. The normality of residuals from these models was studied as aforementioned. When appropriate, a logarithmic transformation was applied to access the normality of dependent variables. For appetite sensations, area under the curves (AUCs) based on trapezoid methods were also analyzed. The tests were two-sided, with a type I error set at 5%. Considering that this study was exploratory, the individual *p*-values were reported without applying systematically mathematical correction but paying particular attention on the magnitude of differences.

## 3. Results

### 3.1. Anthropometric Variables and Body Composition

**Figure 1:**
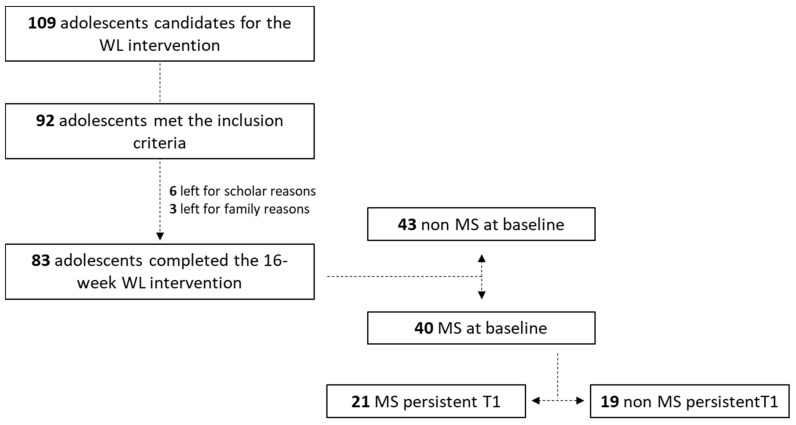
Flow chart of the study.

**Figure 2:**
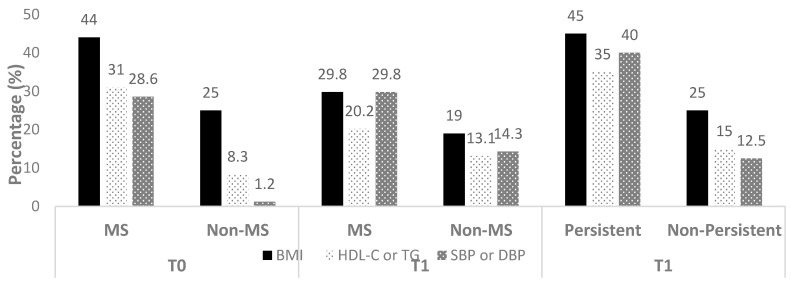
Prevalence of the MS components at baseline and T1 in each group (except for BMI percentile, which remained present in all the adolescents at both times). MS: adolescents with metabolic syndrome; non-MS: adolescents without metabolic syndrome; BMI: body mass index; HDL-C: high density lipoproteins cholesterol; TG: triglycerides; DBP: diastolic blood pressure; SBP: systolic blood pressure; HOMA-IR: homeostatic assessment model for insulin resistance; T0: baseline; T1: post-intervention.

Figure 1 details the flow chart of the study. At baseline, 52.4% of the overall sample was not diagnosed with MS (against 47.6% with MS). Among the 47.6% of the sample who were initially diagnosed with MS at baseline, 52.5% showed persistent MS at the end of the intervention. Figure 2 details the prevalence of each of the MS components (except for BMI, which was present in 100% of the kids at each time and in each group) at baseline and T1 in each subgroups.

As detailed in Table 1, the whole sample showed significant decreases of total body weight (T0: 87.1 ± 14.9 vs. T1: 81.2 ± 13.0 kg; *p* < 0.0001), BMI (T0: 32.9 ± 4.7 vs. T1: 30.4 ± 4.2 kg.m^−2^; *p* < 0.0001), z-BMI (T0: 1.9 ± 0.4 vs. T1: 1.7 ± 0.5; *p* < 0.0001), BMI percentile (T0: 96.2 ± 4.2 vs. T1: 93.8 ± 8.4 kg; *p* < 0.0001), and FM% (T0: 38.1 ± 3.8 vs. T1: 33.8 ± 4.7%; *p* < 0.0001) between baseline (T0) and T1, without significant modification of the adolescents’ FFM. Table 1 also presents the anthropometric and body composition variations in response to the intervention between the adolescents initially diagnosed or not with MS. The MS group showed significantly higher body weight (*p* < 0.001), BMI (*p* < 0.001), z-BMI (*p* < 0.001), as well as FFM (*p* < 0.001) at baseline compared with non-MS. Significant time effects were found for all displayed variables, which decreased in both groups in response to the program (*p* < 0.0001), except for FFM, which remained unchanged *p* = 0.165). Significant group × time interactions were obtained for body weight (*p* = 0.005), BMI (*p* = 0.026), BMI percentile (*p* = 0.031), and FM% (*p* = 0.021), and the MS sample showing greater reductions than that of non-MS in response to the intervention.

Table 2 presents the anthropometric and body composition characteristics between persistent MS vs. non-persistent MS, as well as between non-MS at baseline and non-persistent MS at T1 and their variations in response to the intervention. None of the variable under study was found significantly different at baseline between persistent and non-persistent (data not showed).

Only BMI (*p* = 0.0105) and z-BMI (*p* = 0.02328) were found significantly different between persistent-MS and non-persistent-MS at T1. FM% was found significantly higher among non-MS at T0 (38.0 ± 3.9%) compared with non-persistent at T1 (32.5 ± 42%) (*p* < 0.0001). Inversely, FFM was significantly higher among non-persistent at T1 (54.6 ± 7.4 kg) compared with non-MS at T0 (48.5 ± 7.2 kg) (*p* = 0.003).

### 3.2. Food Consumption and Macronutrient Intake

Table 3 details the whole sample: MS and non-MS energy intake and macronutrient choices at baseline and T1. Ad libitum lunch (T0: 1000 ± 349 kcal vs. T1: 1085 ± 415 kcal; *p* = 0.03) and daily (T0: 1893 ± 548 kcal vs. T1: 1977 ± 523 kcal; *p* = 0.07) increased between baseline and T1 for the whole sample (tendency for daily energy intake). Overall (T0 and T1) lunch and total ad libitum energy intakes were significantly lower among non-MS adolescents (groups effects: *p* < 0.001 and *p* = 0.005, respectively) with a significant time effect for lunch intake only (*p* = 0.027) and no significant interaction. These increases were mainly due to significantly increased absolute protein and absolute as well as relative fat intakes, as detailed in Table 3.

While, as presented in Table 4, there was no significant difference for energy and macronutrient intake between adolescents with persistent and non-persistent MS at T1 (Table 4), lunch and total intake remained significantly higher among adolescents with a non-persistent MS at T1 (lunch: 1214 ± 98 kcal and total: 2110 ± 119 kcal) compared with those who were not diagnosed with the MS at baseline (non-MS at T0; lunch: 885 ± 325 kcal and total: 1735 ± 496 kcal), with *p* = 0.0061 and *p* = 0.0122, respectively. This higher intake in the non-persistent MS T1 sample was mainly explained by higher absolute lunch and total protein intakes (*p* = 0.0014 and *p* = 0.0163, respectively) and higher absolute lunch and total fat intakes (*p* = 0.0025 and *p* = 0.0028, respectively). Significantly higher relative intakes of fat and protein are also observed at lunch in the non-persistent T1 sample (*p* = 0.011 and *p* = 0.0458, respectively) with a lower relative CHO intake (*p* = 0.0441). Similarly, a significantly higher relative total fat intake was observed in the non-persistent T1 group (*p* = 0.0281), with a higher total relative CHO intake (*p* = 0.0149) compared with non-MS at T0 (Table 4).

### 3.3. Appetite Sensations

All the results related to the appetite sensations are presented as additional files. Fasting (T0: 85 ± 111; T1: 88 ± 41 mm; *p* = 0.008) and pre-lunch (T0: 88 ± 42; T1: 97 ± 38 mm; *p* = 0.04) hunger were significantly increased between baseline and T1 when considering the whole sample. Whole sample fasting fullness was significantly decreased (T0: 26 ± 34; T1: 17 ± 25; *p* = 0.04) and PFC increased (T0: 71 ± 40; T1: 83 ± 38; *p* = 0.03) after the intervention. Desire to eat was not significantly modified with the intervention nor the overall daily AUC for the four appetite sensations.

When comparing MS and non-MS adolescents, significant time effects were observed for fasting (*p* = 0.007) and pre-lunch (*p* = 0.047) hunger without group nor interaction effects. Pre-dinner hunger showed a significant group effect (*p* = 0.008), being higher at both baseline and T1 in the MS group (no time nor interaction effects). Fasting fullness and PFC showed significant time effects (*p* = 0.043 and *p* = 0.015 respectively) without group or interaction effects. Pre-dinner PFC showed a significant group effect (*p* = 0.002), being higher at both T0 and T1 in the MS sample (no time nor interaction effects). No significant results were obtained for desire to eat. The overall daily AUC for hunger (*p* = 0.041) and PFC (*p* = 0.013) showed group effects (higher in MS sample) without time and interaction effects.

**Figure 3:**
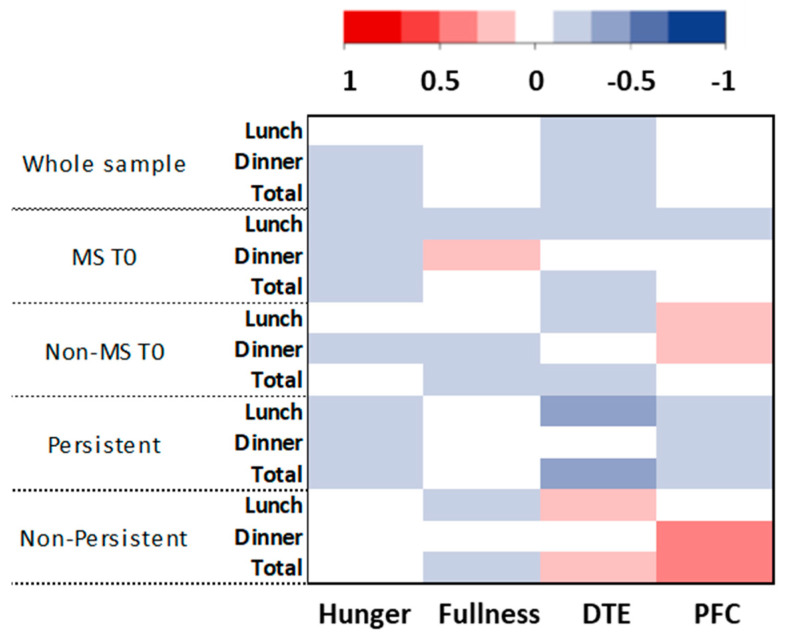
HeatMap representation of the correlations between the baseline and T1 changes of lunch, dinner, and total energy intake and changes of fasting hunger, fullness, desire to eat (DTE), and prospective food consumption (PFC). MS T0: adolescents with the metabolic syndrome at baseline; Non-MS T0: adolescents without the metabolic syndrome at baseline.

None of the appetite sensations under study (time points or AUC) was found different between persistent and non-persistent MS. Fasting hunger (*p* = 0.0297) and pre-dinner hunger (*p* = 0.0175) were both significantly higher in the non-persistent sample compared to values observed at baseline among non-MS adolescents. The other appetite indicators studied were not different between these two subsamples. The detailed appetite results are presented as supplementary files (Tables S1 and S2). Figure 3 illustrates the absence of correlation between changes in fasting appetite sensations and the changes in ad libitum energy intake.

## 4. Discussion

Even though multidisciplinary weight loss interventions are acknowledged for their effectiveness in inducing body weight reduction in adolescents with obesity [24,25], evidence also highlights an important inter-individual variability of this weight loss [8], which, along with the usual post-intervention weight regain, calls for better metabolic phenotyping of adolescents, with a view to both improving our interventions and preventing weight regain. While Miguet et al. have recently reported an increase in ad libitum energy intake after a 10-month multidisciplinary program in adolescents with obesity, they also emphasized huge inter-individual variability of this response and pointed out the need to identify more effectively the adolescents who might experience such a compensatory rise of their food consumption in response to weight loss [8]. Whereas recent evidence shows the associations between the MS and impaired appetite control and eating behavior among adults with obesity and healthy youth [11,12,14], this assertion has so far remained unchallenged among the adolescents with obesity. In that context, the present study compared the energy intake and appetite sensations between adolescents with obesity diagnosed or not with MS and questioned whether the persistence of the MS could be related to adolescents’ appetitive response to weight loss.

First, in line with previous studies conducted in similar populations [8,9,10], our results confirm a significant increase of the adolescents’ food intake in response to the weight loss intervention (ad libitum lunch intake as well as a tendency for total ad libitum energy intake), mainly due to an increased fat and protein intake. Interestingly, this higher energy consumption is accompanied by higher fasting sensations of hunger, prospective food consumption, and a craving to eat, as well as a reduced feeling of fullness. Recent studies have provided similar results in adolescents with obesity in response to a similar 10-month multidisciplinary intervention [8,10], as well as after 12 weeks of both moderate or high-intensity exercise weight loss programs [9].

This increased energy intake at a buffet meal after a weight loss program might represent a sort of loss of control at the abundance of food, which might be deemed a potential indicator of the risk of weight regain upon adolescents’ return to free-living conditions. This increased food consumption, once back in free-living conditions, might be particularly accentuated in these adolescents due to general availability of tasty and energy-dense food items that were not offered in our buffet meal. Regretfully, studies specifically focused on this crucial post-intervention phase are not available, whereas the present findings, along with the previously published ones, definitely merit further research.

The present analysis seems to be the first one in this population focused on comparing appetite control between adolescents with obesity, either affected by MS or not. As evidenced by our results, incidence of MS at this age, on top of obesity, is associated with a higher food intake, mainly due to higher fat and protein ingestion. This significantly higher energy intake is also associated with higher daily (AUC) hunger and prospective food consumption. Despite the scarcity of literature on the effect of the MS on the control of appetite, these results are in line with those obtained by Mirmiran et al., who found an association between unhealthy eating habits and the incidence of MS in children and adolescents, lean or with obesity [12]. This also corroborates previous evidence, thus underlying a positive and significant association between the metabolic profile of adult women with obesity and food craving [11].

As previously referenced, this higher energy consumption in individuals diagnosed with MS might well be accounted for by both altered peripheral [14] and neuro-cognitive (food reward) pathways [13] involved in the control of appetite. Interestingly, whereas the weight loss intervention proved successful in both the MS and non-MS adolescents with regard to anthropometric and body composition variables, in line with the previously published results in similar populations [26], both groups demonstrated an increased energy intake in response to a 16-week multidisciplinary program (no time x group interaction). Interestingly, we also compared these appetitive responses to weight loss between adolescents with persistent and non-persistent MS by the end of the 4 months.

In line with Khammassi et al., who recently reported similar cardio-metabolic responses to a similar weight loss program, irrespective of whether or not the MS remained persistent at the end of the intervention [26], our results demonstrate similar energy intake, macronutrient preference, and overall appetite sensations between persistent and non-persistent MS. Non-enhanced appetite control in response to weight loss, irrespective of whether or not the MS was diagnosed, was also reinforced by the significantly higher energy ingestion observed in the adolescents without persistent MS (non-persistent MS) by the end of the intervention, as compared with the adolescents initially free of the MS. Indeed, the non-persistent MS subsample indicates (T1) a significantly higher lunch and total ad libitum energy intake, mainly through higher fat and protein intakes, when compared to what was observed at baseline in their counterparts unaffected by MS.

Furthermore, the non-persistent MS adolescents demonstrated higher fasting hunger values, as compared to those of the non-MS. In other words, improving adolescents’ MS diagnoses by the end of a weight loss program did not seem to favor enhanced appetite control as compared with the initially metabolically healthy adolescents with obesity (i.e., unaffected by the MS). A potential uncoupled response to a weight loss program between the metabolic profile and appetite control of adolescents with obesity, diagnosed with MS, might then be hypothesized. It highlights the need to more effectively identify and appreciate the underlying mechanisms, as well as map out and promote specifically target-oriented intervention strategies in terms of appetite control.

While the present work is to our knowledge the first to compare energy intake and appetite between adolescents diagnosed or not with MS, questioning the effect of weight loss, its results have to be interpreted in light of some limitations. Firstly, despite making use of an objective and validated methodology for the assessment of energy intake (ad libitum buffet meal) [21], other essential parameters of appetite control might well have also been taken into consideration and duly assessed. The dietary profile of the adolescents, and particularly the level of cognitive restriction, was established to have affected the appetite response to weight loss in this population [8,27]. The present work also certainly lacks the evaluation of some physiological factors that are implicated in the control of appetite. Indeed, it would have been interesting to also evaluate the evolution of the ghrelin and leptin levels, for instance, particularly since their respectively increase [28] and decrease [29] in response to similar weight loss interventions in adolescents with obesity could partly explain our results.

Similarly, since the activity of appetite-related reward system has been found to be associated with the presence and breadth of MS in adults [14], as well as impacted by weight loss in adolescents with obesity [10,30], its evaluation would have been of interest here. Another important limitation to consider remains the dichotomic nature of the actually available diagnosis of the MS [19]. Indeed, as previously pointed out in adults as well as in youth, using strict thresholds to determine the presence of each parameter of MS masks the physiologically continuous nature of these metabolic complications [18]. It would have been also interesting to evaluate the effect of the different combinations of components of the MS on energy intake and appetite sensations, and to question which parameters might be mainly involved in the impaired control of appetite observed. However, the exploratory nature of the present work and its modest sample size did not allow for this analysis. The acute nature of the test meal used in the present study must be considered and longer (over several days) evaluation of the adolescents’ energy intake, in free-living conditions, should be conducted. Moreover, the use of an ad libitum buffet meal might not ideally reflect adolescents’ appetite control and usual energy intake and might be more representative of the degree of their cognitive control (degree of loss of control) in response to abundant food. This is why the selection of the food items was carefully done, avoiding highly palatable items, as detailed in the methods section. Finally, the duration of the study must also be considered when interpreting our results. Indeed, while the present intervention lasted 16 weeks, it has been suggested that adolescents with obesity might continue to adapt their energy intake and appetite control to further weight loss in response to longer interventions [8]. Then this effect of the metabolic syndrome and its persistence would be further questioned in response to longer programs.

To conclude, the present work suggests for the first time impaired appetite control in adolescents with obesity diagnosed with MS compared to similar adolescents without MS. Importantly, the higher energy intake observed despite non-persistent MS compared with persistent MS and initially non-MS adolescents in response to a weight loss intervention might indicate that the treatment of MS in this population might not prevent the adolescents from potential post-intervention compensatory food intake and then weight regain and future potential metabolic disruptions. Further studies exploring the involved physiological and neurocognitive mechanism are needed.

## Figures and Tables

**Table 1:** Whole sample and MS vs. non-MS anthropometric and body composition changed from baseline to T1.

	Whole Sample			MS		Non-MS		Group	Time	Interaction
	T0	T1	*p*	T0	T1	T0	T1			
**Body weight (kg)**	87.1 ± 14.9	81.2 ± 13.0	<0.001	93.3 ± 12.6	86.0 ± 10.6 ***	81.4 ± 14.6 ^c^	76.8 ± 13.6 ***^,b^	<0.000	<0.000	0.005
**BMI (kg.m^−2^)**	32.9 ± 4.7	30.4 ± 4.2	<0.001	34.7 ± 3.8	31.8 ± 3.5 ***	31.2 ± 4.9 ^c^	29.1 ± 4.5 ***^,a^	<0.000	<0.000	0.026
**z-BMI**	1.9 ± 0.4	1.7 ± 0.5	<0.001	2.2 ± 0.3	1.9 ± 0.3 ***	1.7 ± 0.4 ^c^	1.5 ± 0.5 ***^,c^	<0.000	<0.000	0.871
**BMI percentile**	96.2 ± 4.2	93.8 ± 8.4	<0.001	98.1 ± 2.1	96.7 ± 3.2 ***	94.6 ± 5.0	91.1 ± 10.6 ^c^	<0.000	<0.000	0.031
**FM%**	38.1 ± 3.8	33.8 ± 4.7	<0.001	38.3 ± 3.7	33.3 ± 4.4 ***	38.0 ± 3.9	34.3 ± 5.1 ***	0.658	<0.000	0.021
**FFM (kg)**	51.8 ± 8.0	52.1 ± 7.8	0.16	55.5 ± 7.3	55.6 ± 7.1	48.5 ± 7.2 ^c^	48.9 ± 7.2 ^c^	<0.000	0.165	0.528

T0: baseline; T1: end of the intervention; *p*: level of significance; MS: metabolic syndrome at baseline; Non-MS: no metabolic syndrome at baseline; BMI: body mass index; FM%: fat mass percentage; FFM: fat-free mass; ***: *p* < 0.001 between T0 and T1; ^a^: *p* < 0.05 between MS and Non-MS; ^b^: *p* < 0.01 between MS and Non-MS; ^c^: *p* < 0.001 between MS and Non-MS.

**Table 2:** Anthropometric and body composition results in persistent and non-persistent at T1 as well as among non-MS T0 and non-persistent T1.

	Persistent T1	Non-Persistent T1	*p*	Non-MS T0	Non-Persistent T1	*p*
**Body weight (kg)**	88.5 ± 10.3	83.2 ± 10.5	0.116	81.4 ± 14.6	83.2 ± 10.5	0.628
**BMI (kg.m^−2^)**	33.1 ± 3.5	30.3 ± 3.3	0.011	31.2 ± 4.8	30.3 ± 3.3	0.454
**z-BMI**	2.0 ± 1.8	1.8 ± 0.3	0.024	1.7 ± 0.4	1.8 ± 0.3	0.720
**BMI percentile**	97.6 ± 1.4	95.7 ± 4.3	0.076	94.6 ± 5.0	95.7 ± 4.3	0.430
**FM%**	34.0 ± 4.4	32.5 ± 4.2	0.273	38.0 ± 3.9	32.5 ± 4.2	0.000
**FFM (kg)**	56.5 ± 6.8	54.6 ± 7.4	0.386	48.5 ± 7.2	54.6 ± 7.4	0.003

T0: baseline; T1: end of the intervention; *p*: level of significance; Non-MS: no metabolic syndrome at baseline; BMI: body mass index; FM%: fat mass percentage; FFM: fat-free mass.

**Table 3:** Ad libitum energy intake and macronutrient choices at baseline and T1 in the whole sample and among MS and non-MS adolescents.

	Whole Sample			MS		Non-MS		Group	Time	Interaction
	T0	T1	*p*	T0	T1	T0	T1			
**Lunch (kcal)**	1000 ± 349	1085 ± 415	0.03	1125 ± 335	1222 ± 397	885 ± 325	964 ± 396 ^b^	<0.000	0.027	0.473
**Diner (kcal)**	892 ± 286	891 ± 229	0.9	938 ± 312	893 ± 207	850 ± 256	890 ± 249	0.403	0.95	0.19
**Total ad libitum (kcal)**	1893 ± 548	1977 ± 523	0.07	2063 ± 557	2116 ± 502	1735 ± 496	1854 ± 516	0.005	0.081	0.842
**Protein Lunch (g)**	49 ± 18	54 ± 22	0.004	57 ± 17	63 ± 18 ^b^	42 ± 16 ^b^	47 ± 21	<0.000	0.004	0.724
**Fat lunch (g)**	27 ± 17	29 ± 19	0.04	35 ± 16	37 ± 17	19 ± 13 ^c^	22 ± 18 ^c^	<0.000	0.042	0.934
**CHO lunch (g)**	137 ± 55	147 ± 73	0.18	143 ± 54	154 ± 78	131 ± 56	141 ± 68	0.242	0.174	0.52
**Protein dinner (g)**	54 ± 24	54 ± 24	0.71	55 ± 25	52 ± 19	53 ± 24	57 ± 27	0.848	0.758	0.097
**Fat dinner (g)**	28 ± 15	27 ± 12	0.78	32 ± 17	30 ± 13	26 ± 13	25 ± 11	0.074	0.768	0.742
**CHO dinner (g)**	105 ± 34	105 ± 29	0.87	107 ± 37	103 ± 25	104 ± 30	107 ± 32	0.979	0.835	0.329
**Total Protein (g)**	104 ± 32	109 ± 33	0.03	113 ± 33	115 ± 30	95 ± 28	104 ± 34	0.030	0.032	0.393
**Total Fat (g)**	56 ± 27	57 ± 25	0.26	67 ± 28	67 ± 23	45 ± 21	47 ± 22	<0.000	0.271	0.773
**Total CHO (g)**	242 ± 76	253 ± 87	0.29	250 ± 75	258 ± 89	235 ± 76	248 ± 86	0.376	0.292	0.934
**Protein Lunch (%)**	19.6 ± 3.8	20.3 ± 4.2	0.06	20.5 ± 3.4	20.8 ± 3.0	18.8 ± 3.9	19.8 ± 5.0	0.102	0.060	0.28
**Fat lunch (%)**	23.1 ± 11.4	23.4 ± 11.2	0.55	27.8 ± 10.8	27.6 ± 9.8	18.7 ± 10.3 ^b^	19.6 ± 11.1 ^b^	<0.000	0.561	0.377
**CHO lunch (%)**	55.5 ± 14.4	55.2 ± 14.3	0.54	51.6 ± 12.7	50.5 ± 12.2	59.3 ± 14.7	59.5 ± 14.8 ^a^	0.008	0.600	0.527
**Protein dinner (%)**	24.6 ± 9.5	24.2 ± 7.3	0.51	23.7 ± 7.8	23.5 ± 7.1	25.6 ± 10.7	24.8 ± 7.5	0.394	0.583	0.678
**Fat dinner (%)**	30.7 ± 23.1	28.2 ± 10.6	0.36	30.4 ± 10.7	30.0 ± 9.6	31.5 ± 30.7	26.6 ± 11.2	0.781	0.428	0.393
**CHO dinner (%)**	49.6 ± 24.3	47.6 ± 8.2	0.31	46.1 ± 10.0	46.6 ± 6.7	53.4 ± 32.0	48.4 ± 9.2	0.201	0.376	0.388
**Total Protein (%)**	22.1 ± 3.6	22.2 ± 3.3	0.68	22.0 ± 3.3	21.8 ± 2.7	22.1 ± 4.0	22.5 ± 3.7	0.516	0.714	0.355
**Total Fat (%)**	26.2 ± 8.8	26.1 ± 8.8	0.63	29.1 ± 8.4	29.1 ± 7.8 ***	23.5 ± 8.2	23.4 ± 8.8 ***^,c^	0.003	0.636	0.973
**Total CHO (%)**	51.9 ± 9.4	51.1 ± 9.2	0.18	48.9 ± 8.4	48.5 ± 8.1 ***	54.6 ± 9.5	53.4 ± 9.5 ***	0.626	<0.000	0.274

T0: baseline; T1: end of the intervention; *p*: level of significance; MS: metabolic syndrome at baseline; Non-MS: no metabolic syndrome at baseline; CHO: carbohydrate; ***: *p* < 0.001 between T0 and T1; ^a^: *p* < 0.05 between MS and Non-MS; ^b^: *p* < 0.01 between MS and Non-MS; ^c^: *p* < 0.001 between MS and Non-MS.

**Table 4:** Ad libitum energy intake and macronutrient choices in persistent vs. non-persistent adolescents at T1 and among non-MS T0 and non-persistent at T1.

	Persistent T1	Non-Persistent T1	*p*	Non-MS T0	Non-Persistent T1	*p*
**Lunch (kcal)**	1229 ± 84	1214 ± 98	0.907	885 ± 325	1214 ± 98	0.0061
**Diner (kcal)**	891 ± 49	895 ± 45	0.952	850 ± 256	895 ± 45	0.4535
**Total ad libitum (kcal)**	2121 ± 111	2110 ± 119	0.946	1735 ± 496	2110 ± 119	0.0122
**Protein Lunch (g)**	62 ± 3	63 ± 5	0.895	42 ± 16	63 ± 5	0.0014
**Fat lunch (g)**	37 ± 3	37 ± 5	0.921	19 ± 13	37 ± 5	0.0025
**CHO lunch (g)**	157 ± 17	151 ± 18	0.821	131 ± 56	151 ± 18	0.3163
**Protein dinner (g)**	50 ± 4	54 ± 5	0.508	53 ± 24	54 ± 5	0.7597
**Fat dinner (g)**	30 ± 3	30 ± 2	0.961	26 ± 13	30 ± 2	0.2442
**CHO dinner (g)**	104 ± 5	101 ± 6	0.692	104 ± 30	101 ± 6	0.7315
**Total Protein (g)**	113 ± 28	118 ± 33	0.612	95 ± 28	118 ± 33	0.0163
**Total Fat (g)**	67 ± 21	67 ± 25	0.96	45 ± 21	67 ± 25	0.0028
**Total CHO (g)**	262 ± 19	253 ± 21	0.756	235 ± 76	253 ± 21	0.4697
**Protein Lunch (%)**	20.8 ± 0.5	21.0 ± 0.8	0.826	18.8 ± 3.9	21.0 ± 0.8	0.0458
**Fat lunch (%)**	27.7 ± 1.7	27.5 ± 2.8	0.953	18.7 ± 10.3	27.5 ± 2.8	0.011
**CHO lunch (%)**	50.5 ± 2.1	50.5 ± 3.5	0.997	59.3 ± 14.7	50.5 ± 3.5	0.0441
**Protein dinner (%)**	22.7 ± 1.4	24.6 ± 1.8	0.43	25.6 ± 10.7	24.6 ± 1.8	0.6694
**Fat dinner (%)**	29.8 ± 2.0	30.2 ± 2.4	0.886	31.5 ± 30.7	30.2 ± 2.4	0.8134
**CHO dinner (%)**	47.8 ± 1.5	45.3 ± 1.6	0.252	53.4 ± 32.0	45.3 ± 1.6	0.1221
**Total Protein (%)**	21.4 ± 0.5	22.3 ± 0.7	0.306	22.1 ± 4.0	22.3 ± 0.7	0.8523
**Total Fat (%)**	28.9 ± 1.4	29.3 ± 2.2	0.865	23.5 ± 8.2	29.3 ± 2.2	0.0281
**Total CHO (%)**	49.2 ± 1.5	47.8 ± 2.2	0.604	54.6 ± 9.5	47.8 ± 2.2	0.0149

T0: baseline; T1: end of the intervention; *p*: level of significance; Non-MS: no metabolic syndrome at baseline; CHO: carbohydrate.

## Supplementary Materials

The following are available online at https://www.mdpi.com/2072-6643/12/12/3885/s1, Table S1: Appetite sensation between T0 and T1 for the whole sample and MS and non-MS subsamples, Table S2: Appetite sensation between Persistent vs. non-persistent adolescents at T1 and between non-MS T0 and non-Persistent at T1.
