# Supplementary material for: Appetite Control Might not Be Improved after Weight Loss in Adolescents with Obesity, Despite Non-Persistent Metabolic Syndrome
Source: Nutrients. 2020 Dec 18;12(12):3885. doi: 10.3390/nu12123885 (PMC7766326; doi:10.3390/nu12123885)
Supplement: Supplementary file 1 [file nutrients-12-03885-s001.pdf]

Additional files.

Table S1. Additional files. Appetite sensation between T0 and T1 for the whole sample and MS and non-MS subsamples.

	Whole sample			MS		Non-MS		*Group*	*Time*	*Interaction*
	T0	T1	p	T0	T1	T0	T1			
**Fasting Hunger (mm)**	85 ± 111	88 ± 41	0,008	94 ± 24	88 ± 42	75 ± 38	88 ± 40	0,828	0,007	0,680
**Pre Lunch Hunger (mm)**	88 ± 42	97 ± 38	0,04	96 ± 6	102 ± 38	80 ± 6	93 ± 37	0,109	0,047	0,600
**Pre Diner Hunger (mm)**	87 ± 38	92 ± 34	0,22	95 ± 6	102 ± 31	79 ± 5	83 ± 35	0,008	0,211	0,515
**Fasting Fullness (mm)**	26 ± 34	17 ± 25	0,04	20 ± 4	16 ± 24	31 ± 6	17 ± 25	0,409	0,043	0,305
**Pre Lunch Fullness (mm)**	25 ± 34	25 ± 35	0,98	26 ± 5	27 ± 37	24 ± 5	23 ± 33	0,226	0,977	0,701
**Pre Diner Fullness (mm)**	30 ± 37	28 ± 39	0,65	26 ± 4	28 ± 40	34 ± 6	28 ± 39	0,868	0,719	0,404
**Fasting PFC (mm)**	71 ± 40	83 ± 38	0,03	74 ± 6	89 ± 37	69 ± 6	78 ± 39	0,347	0,015	0,513
**Pre Lunch PFC (mm)**	83 ± 40	91 ± 40	0,08	92 ± 6	99 ± 37	73 ± 6	85 ± 42	0,062	0,043	0,610
**Pre Diner PFC (mm)**	87 ± 38	85 ± 39	0,67	100 ± 5	96 ± 36	75 ± 5a	76 ± 39	0,002	0,660	0,805
**Fasting DTE (mm)**	94 ± 36	105 ± 40	0,07	88 ± 7	104 ± 40	103 ± 5	106 ± 42	0,555	0,095	0,373
**Pre Lunch DTE (mm)**	105 ± 36	106 ± 37	0,59	107 ± 7	108 ± 36	103 ± 7	102 ± 39	0,486	0,564	0,738
**Pre Diner DTE (mm)**	99 ± 39	93 ± 46	0,57	104 ± 6	101 ± 44	94 ± 9	83 ± 47	0,085	0,558	0,889
**AUC Hunger (mm)**	48687 ± 21101	52627 ± 17,015	0,09	53589 ± 21540	56313 ± 16061	45062 ± 20742	49530 ± 17272	0,041	0,094	0,940
**AUC Fullness (mm)**	30420 ± 18053	29741 ± 16798	0,76	31451 ± 17642	27795 ± 17132	30425 ± 19419	32533 ± 17511	0,195	0,786	0,329
**AUC PFC (mm)**	49822 ± 19099	51486 ± 17523	0,46	54979 ± 17510	47459 ± 18865	43419 ± 21624	55669 ± 14585	0,013	0,466	0,884
**AUC DTE (mm)**	58273 ± 17765	57445 ± 18156	0,75	61173 ± 16022	52932 ± 21230	54733 ± 19616	60414 ± 14766	0,106	0,742	0,854

T0: Baseline; T1: end of the intervention; p: level of significance; MS: Metabolic syndrome at baseline; Non-MS: No Metabolic syndrome at baseline; PFC: Prospective Food Consumption; DTE: Desir To Eat; ***: p<0.001 between T0 and T1; a: p<0.05 between MS and Non-MS; b: p<0.01 between MS and Non-MS; c: p<0.001 between MS and Non-MS.

Table S2. Additional files. Appetite sensation between Persistent vs. non-persistent adolescents at T1 and between non-MS T0 and non-Persistent at T1.

	Persistent	Non-Presistent	p	Non-MS T0	Non-Persistent T1	p
**Fasting Hunger (mm)**	78 ± 9	100 ± 9	0,1029	75 ± 38	100 ± 9	0.029
**Pre Lunch Hunger (mm)**	110 ± 7	93 ± 10	0,1685	80 ± 6	93 ± 10	0.300
**Pre Diner Hunger (mm)**	104 ± 7	100 ± 6	0,6853	79 ± 5	100 ± 6	0.017
**Fasting Fullness (mm)**	15 ± 5	18 ± 6	0,7193	31 ± 6	18 ± 6	0.141
**Pre Lunch Fullness (mm)**	23 ± 7	32 ± 9	0,4864	24 ± 5	32 ± 9	0.442
**Pre Diner Fullness (mm)**	27 ± 9	29 ± 9	0,8990	34 ± 6	29 ± 9	0.647
**Fasting PFC (mm)**	86 ± 7	92 ± 9	0,6844	69 ± 6	92 ± 9	0.060
**Pre Lunch PFC (mm)**	107 ± 7	89 ± 9	0,1326	73 ± 6	89 ± 9	0.184
**Pre Diner PFC (mm)**	101 ± 8	90 ± 8	0,3369	75 ± 5	90 ± 8	0.161
**Fasting DTE (mm)**	100 ± 8	110 ± 11	0,5092	103 ± 5	110 ± 11	0.591
**Pre Lunch DTE (mm)**	121 ± 4	90 ± 12	0,0310	103 ± 7	90 ± 12	0.399
**Pre Diner DTE (mm)**	105 ± 9	94 ± 12	0,4897	94 ± 9	94 ± 12	0.887
**AUC Hunger (mm)**	58781 ± 16390	53435 ± 15630	0,306	45062 ± 20742	53435 ± 15630	0,13
**AUC Fullness (mm)**	33242 ± 18126	31750 ± 17265	0,792	30425 ± 19419	31750 ± 17265	0,80
**AUC PFC (mm)**	57865 ± 16404	52956 ± 11893	0,309	43419 ± 21624	52956 ± 11893	0,09
**AUC DTE (mm)**	63332 ± 13290	56150 ± 16283	0,181	54733 ± 19616	56150 ± 16283	0,83

T0: Baseline; T1: end of the intervention; p: level of significance; Non-MS: No Metabolic syndrome at baseline; PFC: Prospective Food Consumption; DTE: Desir To Eat
